# Interaction Network of Proteins Associated with Human Cytomegalovirus IE2-p86 Protein during Infection: A Proteomic Analysis

**DOI:** 10.1371/journal.pone.0081583

**Published:** 2013-12-16

**Authors:** Guixin Du, Mark F. Stinski

**Affiliations:** Department of Microbiology, Carver College of Medicine, University of Iowa, Iowa City, Iowa, United States of America; National Institute of Health - National Cancer Institute, United States of America

## Abstract

Human cytomegalovirus protein IE2-p86 exerts its functions through interaction with other viral and cellular proteins. To further delineate its protein interaction network, we generated a recombinant virus expressing SG-tagged IE2-p86 and used tandem affinity purification coupled with mass spectrometry. A total of 9 viral proteins and 75 cellular proteins were found to associate with IE2-p86 protein during the first 48 hours of infection. The protein profile at 8, 24, and 48 h post infection revealed that UL84 tightly associated with IE2-p86, and more viral and cellular proteins came into association with IE2-p86 with the progression of virus infection. A computational analysis of the protein-protein interaction network indicated that all of the 9 viral proteins and most of the cellular proteins identified in the study are interconnected to varying degrees. Of the cellular proteins that were confirmed to associate with IE2-p86 by immunoprecipitation, C1QBP was further shown to be upregulated by HCMV infection and colocalized with IE2-p86, UL84 and UL44 in the virus replication compartment of the nucleus. The IE2-p86 interactome network demonstrated the temporal development of stable and abundant protein complexes that associate with IE2-p86 and provided a framework to benefit future studies of various protein complexes during HCMV infection.

## Introduction

Human cytomegalovirus (HCMV), a prototype β-herpesvirus, causes life-threatening disease in immunocompromised adults, such as AIDS patients and organ transplant recipients, whereas it usually causes asymptomatic persistent infection in healthy individuals. In addition, it is the leading infectious cause of congenital abnormalities and mental retardation in newborns in the United States [1]. Furthermore, chronic HCMV infection has recently been implicated as a cofactor in cardiovascular disease [2] as well as malignant diseases [2]–[4].

HCMV only infects humans and replicates preferentially in terminally differentiated cells. Infection progresses through three temporal phases, defined as immediate early (IE), early (E), and late (L). Transcription of the IE genes occurs at five genetic loci and is independent of *de novo* viral protein synthesis. IE gene products have multiple functions including activating expression of early viral genes, inhibiting apoptosis, and countering intrinsic and innate host immunity [5], [6]. Early viral proteins either participate directly in viral DNA synthesis or provide an optimal cellular condition for viral DNA replication. The late genes, which primarily encode structural proteins, are expressed after viral DNA replication [1].

The major immediate-early (MIE) gene locus, a master switch for lytic HCMV infection, generates two predominant viral proteins, IE1-p72 and IE2-p86, and several minor isoforms [6]. While the most abundant MIE protein, IE1-p72, is only required for HCMV replication at low multiplicity of infection (MOI), the less abundant IE2-p86 is essential for viral replication [7], [8]. IE2-p86 protein has been extensively studied using *in vitro* methods and multiple functions have been ascribed to it. IE2-p86 binds to a 14-base pair *cis*-repression sequence in the MIE promoter to negatively autoregulate expression of the IE1 and IE2 transcripts [9]–[11]. It transactivates viral early gene expression via its interactions with cellular basal transcription machinery [12]. It up-regulates a vast array of cellular genes, including those involved in progression of the cell cycle from G_0_/G_1_ to S phase, such as the E2F-responsive genes [13]. However, the IE2-p86 protein also arrests cell cycle progression in both p53-wild type and -null cells [6], [14], [15]. Moreover, the IE2-p86 protein can block virus-induced chemokine expression [16].

A collection of published reports indicates that IE2-p86 protein can bind to a wide array of different cellular proteins, including pRb, p53, p21, Cdt1, basal transcription factors (TBP, TFIIB, and TAFs), histone acetylases (CBP, p300, and PCAF), histone deacetylases (HDAC1, HDAC2, and HDAC3), histone methyltransferases (G9a and Suvar(3-9)H1), SUMO-1 and Ubc9, mdm2, PIAS1, and Sp1 (reviewed in Stinski and Petrik, 2008) [6]. Although these studies provide insight into the mechanism of IE2-p86 function, many of the studies are limited in interaction by extrapolation from results of *in vitro* binding assays or the forced over-expression of proteins of interest. Nevertheless, IE2-p86 likely exerts many of its biological functions by way of stable as well as transitory protein-protein interactions. There remains a major gap in knowledge as to the temporal sequence of these interactions and which proteins bind to IE2-p86 under normal infected cell conditions.

Developments in affinity-purification based isolation methods coupled with mass spectometry (AP-MS) has greatly facilitated identification of proteins in isolated complexes [17]. For example, over 50 cellular proteins were identified to interact with herpes simplex virus early protein ICP8 [18]. The ICP8 interactome is involved in various cellular functions such as viral DNA replication, DNA repair, recombination, and chromatin re-modeling. With HCMV, the interacting partners of viral proteins UL84, UL44, UL38, UL29/28, and UL35/35a have been studied using the AP-MS method [19]–[24]. IE2-p86 binds to itself and to the viral protein UL84 to form a complex involved in the initiation of viral DNA synthesis from oriLyt [25]. Gao et al. reported that viral protein UL84 interacts with cellular protein ubiquitin-conjugating enzyme E2, casein kinase II, p32 (C1QBP), and importin, as well as viral proteins UL44 and pp65 [24]. Strang et al. detected nucleolin, UL54, IRS1, and UL25 associated with UL44 during the late phase of infection with HCMV [22]. Given the approximately 175 designated open reading frames (ORF) of HCMV and, the approximately 751 putative ORFs identified recently [26], there is much to be learned about the HCMV interactome.

In this study, we used tandem affinity purification- mass spectrometry (TAP-MS) to identify proteins that stably associate with IE2-p86 protein in HCMV-infected cells at various times after infection. A total of 9 viral proteins and 75 cellular proteins were found to associate with IE2-p86 protein during the first 48 hours of infection. The viral proteins IE2-p86 and UL84 formed a dominant complex very early after infection. The protein-protein interacting network generated by computational analysis demontrates the interconnection of all 9 viral proteins and the majority of the identified cellular proteins. We analyzed one of the viral-cellular protein complexes in more detail. Cellular protein C1QBP interacted with the IE2-p86 early after infection and was upregulated by HCMV infection. C1QBP colocalized with IE2-p86, UL84 and UL44 in the virus replication compartments of the nucleus.

## Materials and Methods

### Human Foreskin Fibroblast Cells (HFF)

Anonymous samples of human foreskins to prepare primary fibroblast cells were obtained from the University of Iowa Hospital and Clinics. The University of Iowa Institutional Review Board waived the need to obtain informed consent for obtaining normally discarded human foreskin tissues that could not be linked to private identifiable information according to protocols approved by the Institutional Review Board. HFF cells were isolated and grown in Eagle's minimal essential medium (MEM; Mediatech, Herndon, VA) supplemented with 10% newborn calf serum (Sigma, St. Louis, MO), as described previously [27].

### Plasmids, BACs, and recombinant viruses

**Figure 1:**
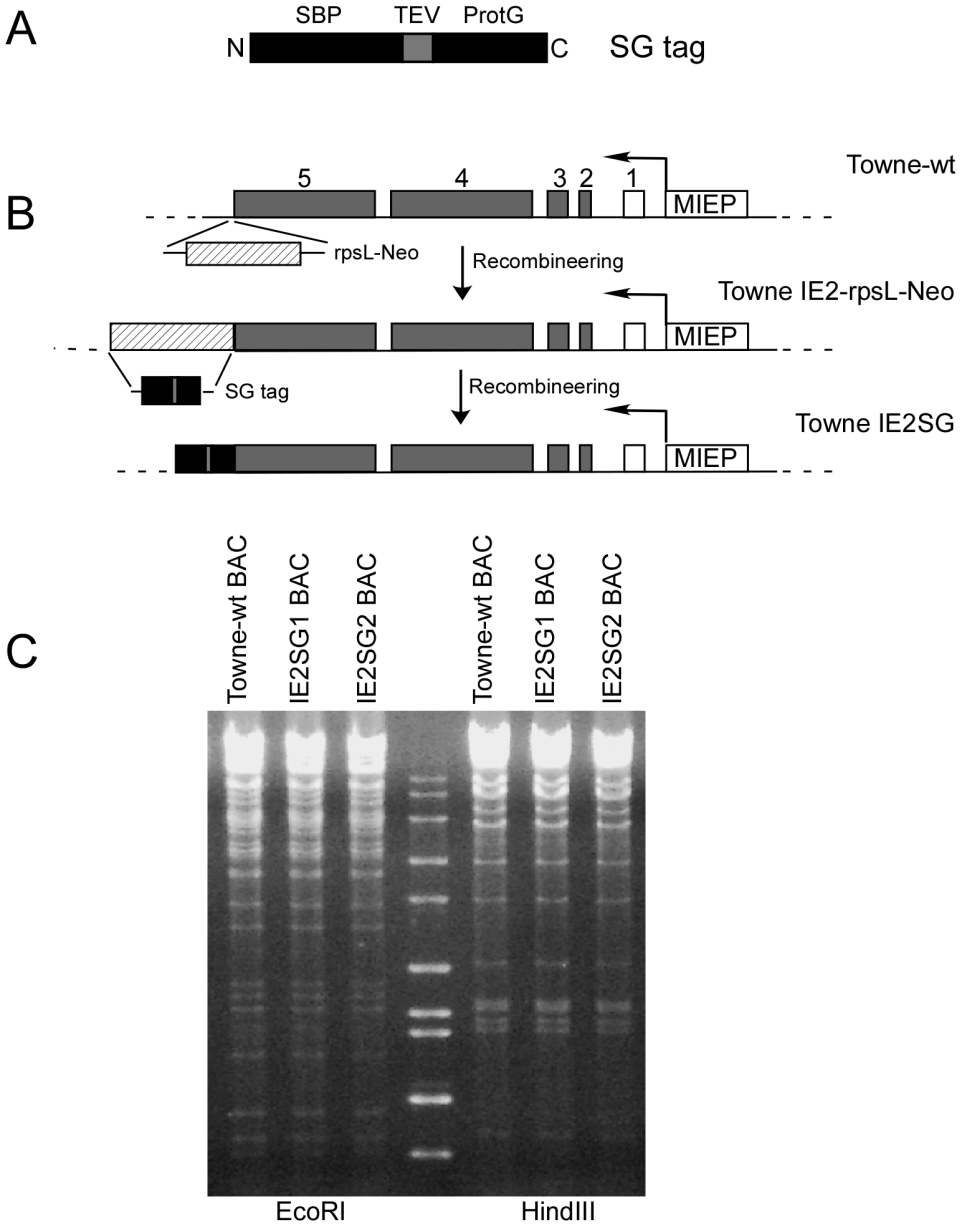
Recombinant HCMVs expressing IE2-p86 protein with a Carboxyl-terminal SG-tag. (A) At the amino terminus (N) of the tag is the streptavidin binding peptide (SBP) followed by the Tobacco Etch Virus (TEV) protease cleavage site and at the carboxyl terminus (C) is the protein G peptide (ProtG) that binds to IgG. (B) Schematic representation of the HCMV MIE gene locus downstream of the MIE promoter (MIEP) of wt Towne BAC. During the first recombination step, the Rspl-Neo cassette was inserted at the 3′ end of MIE Exon 5 to render DY380 *E. coli* kanamycin resistant and streptomycin sensitive. The Rspl-Neo was replaced by the SG-tag during the second recombination step as described in the Materials and Methods. (C) Recombinant BAC DNAs digested with restriction endonucleases EcoRI or HindIII and compared to wt HCMV BAC DNA after agarose gel electrophoresis.

The plasmid pCeMM-CTAP(SG), containing tandem affinity purification SG-tag that consists of Streptavidine Binding Peptide (SBP), the tobacco etch virus (TEV) protease cleavage sites, and two copies of Protein G peptide (ProtG, gift from EUROSCARF, Frankfurt Germany) is diagramed in Figure 1A and described previously [28]. The SG-tag sequence was inserted at the 3′ end of MIE Exon 5 in wildtype Towne-BAC [29]. A two-step recombination method based on Rspl-Neo counter-selection (Gene Bridges GmbH, Heidelberg, Germany) in *Escherichia coli* strain DY380 was used as described previously [30]. PCR primer pair Exon5rspl (Table 1) was used to amplify the DNA template from plasmid pRspl-Neo consisting of the *rspL* and kanamycin resistance genes flanked on either side by 50-base pairs (bp) of homologous sequence around the MIE Exon 5 3′ terminus. This PCR product was electroporated into DY380 harboring wt Towne BAC DNA with GFP inserted into the viral genome for first stage recombination. Colonies were screened by PCR (primers Exon5-seq forward and rpsl-seq reverse, see table 1) and by restriction fragment length polymorphism (RFLP) analyisis with restriction endonuclease EcoRI or HindIII. Two independent BAC clones containing rspl-Neo cassette were selected for second stage recombination. A DNA fragment with SG-tag sequence flanked on either side by the same 50-bp of homologous sequence around the MIE Exon 5 3′ terminus was generated by PCR amplification of plasmid pCeMM-CTAP with primer pair Exon5CTAP (Table 1). This PCR product was then electroporated into DY380 harboring intermediate recombinant Towne BAC for replacing rspl-neo cassette with SG tag as diagram in figure 1A. After screening by colony PCR and RFLP analysis with restriction endonuclease EcoR I and Hind III, mutant BACs with the desired SG tag were confirmed by DNA sequencing. Two independently selected BACs, designated as IE2SG1 and IE2SG2, were used for this investigation.

**Table 1:** Oligos used for fusing SG to the C-terminus of HCMV IE2 and for diagonostics.

Primer name	Direction^a^	Sequence (5′ to 3′)
Exon5CTAP^b^	Forward	TGAGCCTGGCCATCGAGGCAGCCATCCAGGACCTGAGGAACAAGTCTCAG **CCGGCCGAGCAGAAGCTTATCTC**
	Reverse	ACGGGGAATCACTATGTACAAGAGTCCATGTCTCTCTTTCCAGTTTTTCAC **TTATTCAGTGACAGTGAAAGTCT**
Exon5Rpsl^c^	Forward	AGCCTGGCCATCGAGGCAGCCATCCAGGACCTGAGGAACAAGTCTCAGTAA ***GGCCTGGTGATGATGGCGGGATC***
	Reverse	ACGGGGAATCACTATGTACAAGAGTCCATGTCTCTCTTTCCAGTTTTTCAC ***TCAGAAGAACTCGTCAAGAAGG***
Exon5-seq	Forward	GGGTGGGTTCATGCTGCCTATCT
	Reverse	AGCGGGGTAACCAAAGAAATCACA
Rpsl-seq	Forward	GGCCTGGTGATGATGGCGGGATC
	Reverse	TCAGAAGAACTCGTCAAGAAGG

^a^ Directionality of the primer.

^b^ The underlined nucleotides are the IE2-p86-specific sequences, boldface nucleotides represent priming sites within the mutagenesis template plasmid pCeMM-CTAP.

^c^ The underlined nucleotides are the IE2-p86-specific sequences, boldface nucleotides represent priming sites within the template plasmid pRpsl-Neo.

Recombinant HCMV BAC viruses were reconstituted and propagated in human foreskin fibroblast (HFF) cells as described previously [31]. The HFF cells were transfected with either 1 or 3 µg of each recombinant HCMV BAC and 1 µg of the plasmid pSVpp71 using the calcium phosphate precipitation method. Extracellular fluid was harvested 5 to 7 days after 100% cytopathic effect (CPE) and stored at −80°C in 50% newborn calf serum. Virus titers were determined on HFF cells by counting green fluorescent plaque forming units (PFU/ml) at 6 days post infection as described previously [32].

### Tandem affinity purification

HFF cells (≥10^8^ cells/purification) were infected in parallel with either wt Towne or Towne IE2SG at a MOI of 2 PFU/cell. At 8, 24, and 48 h post infection (p.i.), cells were harvested for tandem affinity purification (TAP) as described previously [28]. Briefly, monolayer cells were harvested by trypnization, washed with phosphate buffered saline (PBS), and resuspened in lysis buffer (50 mM Tris-HCl pH 7.5, 125 mM NaCl, 5% glycerol, 0.5% NP-40, 1.0 mM MgCl_2_, 25 mM NaF, 1 mM Na_3_VO_4_, 1× protease inhibitor cocktail) on ice for 30 min. The lysate, cleared by centrifugation, was incubated at 4°C for 2 h with rabbit-IgG agarose (A2909, Sigma-Aldrich) in the presense of Benzonase ® nuclease (10 U/ml, Novagen). The bound proteins were washed with lysis buffer three times followed by one wash with TEV-protease cleavage buffer (10 mM Tris-HCl [pH 7.5], 100 mM NaCl and 0.2% NP-40) and eluted by addition of 100 U of TEV protease (Invitrogen) at 4°C overnight. The TEV-protease cleavage product was then incubated at 4°C for 2 h with Ultralink Immobilized Streptavidin Plus resin (Pierce, Rockford, IL). After extensive washing with TEV-protease cleavage buffer, the bound proteins were eluted with 2 mM D-biotin buffer (Invitrogen). To assess the efficiency of the biotin elution, we boiled the remaining streptavidin beads in SDS-containing sample buffer and analyze the eluate by SDS-PAGE and silver staining.

### Sample preparation

One tenth (1/10) of the D-biotin eluate was fractionated by SDS-PAGE in a 4–12% gel (Invitrogen) and the polypeptides were visualized by silver staining (SilverQuest, Invitrogen). The remaining eluate was concentrated in a ultrafiltration column (YM-10, Microcon), reduced with DTT, alkylated with iodoacetamide (Pierce), and digested in solution with modified porcine trypsin (Promega Corp., Madison, WI) at 37°C overnight, as described previously [33]. Prior to analysis by nanoLC-MS/MS, tryptically-digested samples were purified and concentrated by using UltraMicroSpin reversed-phase columns (The Nest Group Inc. Southboro, MA).

### Mass spectrometry and data analysis

The samples were analysed by data-dependent nanocapillary reversed-phase LC-MS/MS using a C18 column on a nanoLC system (Agilent Technologies, Palo Alto, CA) coupled to a Thermo LTQ-XL linear ion trap mass spectrometer. Data-dependent acquisition was performed for 75 min using one MS channel for every three MS/MS channels and a permanent exclusion list of the most frequent peptide contaminants (keratins and trypsin peptides) was included in the acquisition method to focus the analysis on significant data.

All data generated by LC–MS/MS, were searched against human SwissProt protein database (SwissProt_2011_07), appended with proteins from other herpes viruses, and HCMV (total 120914 entries) by both Mascot (Matrix Science, London, UK; version 2.3 Mascot) and X! Tandem (GPM, version 2010.01.01). One missed tryptic cleavage site was allowed. Mascot and X! Tandem were searched with a fragmention mass tolerance of 0.80 Da and a parent ion tolerance of 1.5 Da. Iodoacetamide derivative of cysteine was specified as a fixed modification in Mascot and X! Tandem. Deamination of asparagines and glutamines, oxidation of methionines and iodoacetamide derivatives of the N-terminus were set as variable modifications in Mascot and X! Tandem. Search results from Mascot and X! Tandem were imported into Scaffold (version Scaffold_3.3.3, Proteome Software Inc., Portland, OR) to validate the MS/MS based peptide and protein identifications. Peptide identifications were accepted if they could be established at greater than 95.0% probability as specified by the Peptide Prophet algorithm [34]. Protein identifications were accepted if they could be established at greater than 99.0% probability and contained at least 2 identified peptides. Protein probabilities were assigned by the Protein Prophet algorithm [35]. Proteins that contained similar peptides and could not be differentiated based on MS/MS analysis alone were grouped to satisfy the principles of parsimony. To assess the incidence of false positive identifications, the data were also searched against an inverted tryptic peptide database, i.e., a theoretical tryptic digest of the database was performed and for each tryptic peptide the sequence of the peptide was reversed but still maintained a tryptic peptide identity (inverted peptides terminate in a lysine or arginine residue). A false-positive detection rate of less than 1% on the protein groups was imposed. Proteins that appeared in control data sets or were common contaminants from MS data reported in the literature [36] were not accepted as potential IE2-p86 interacting proteins.

After removing the commonly reported contaminants in MS/MS, the protein complexes associated with IE2-p86 were analyzed using online database resource Search Tool for the Retrieval of Interacting Genes (STRING, Ver. 9.0). The protein-protein interaction results of *in silico* networking analysis from STRING database combined with those demonstrated experimentally in the literature were visualized by Cytoscape software (version 2.8.3). Solid lines show protein–protein physical/functional interactions based on experimental evidence from the literature and this study, while dashed lines indicate protein–protein interactions suggested by STRING database using predictive methods, such as textmining, coexpression, neighborhood, and co-occurance [37]. To simplify the map, only representative ribosomal proteins were shown.

### Western blot

Cells were harvested, lysed and processed for western blot analysis as described previously [27]. Cell lysates were fractionated in 10% SDS-PAGE gel. Proteins were transferred to a polyvinylidene difluoride (PVDF) membranes, and were probed with the following antibodies: anti-MIE MAB 810 (Chemicon, Temecula, CA), anti-IE2-p86 (12E2, sc-69835, Santa Cruz Biotechnology, Santa Cruz, CA), anti-IE1 exon4 p63-27 (a kind gift from W. Britt, University of Alabama, Birmingham), anti-UL44 MAb (DakoCytomation, Carpinteria, CA), anti-UL84 (Mab84, Santa Cruz), anti-UL99 MAb (Fitzgerald, Concord, MA), anti-UL83 (Fitzgerald, Concord, MA), anti-GAPDH (Chemicon), rabbit anti-C1QBP polyclonal antibody (sc-48795, Santa Cruz), rabbit anti-PTRF polyclonal antibody (ab48824, Abcam, MA), anti-NPM1 McAb (sc-32256, Santa Cruz) and rabbit anti-YB1 polyclonal antibody (ab12148, Abcam). Secondary antibodies used in the study were HRP-conjugated goat anti-mouse immunoglobulin G (IgG) or secondary HRP-conjugated donkey anti-rabbit IgG. SuperSignal West Pico chemiluminescence detection reagent (Pierce, Rockford, IL) was used for developing signal from samples according to the manufacturer's instructions.

For cell fractionation, mock-and HCMV-infected HFF cells were harvested at 2 d p.i. by trysinization. Cell pellets were washed with 1× PBS, resuspended in 2 ml of ice-cold hypotonic buffer (10 mM HEPES, pH 7.9, 1.5 mM MgCl_2_, 10 mM KCl, 1 mM DTT, and 1× protease inhibitor) and kept on ice for 5 min. Cells were broken open using a pre-chilled Dounce homogenizer to release nuclei. The dounced cells were centrifuged at 300*×* g for 5 min at 4°C to pellet nuclei. The supernatant was retained as the cytoplasmic fraction and the pellet was nuclear fraction. Both cytoplasmic and nuclear fraction were processed in 5X SDS-PAGE sample buffer and applied to western blot analysis. Lamin A and GAPDH were detected as marker of nuclear fraction and cytoplasmic fractions, respectively, with corresponding monoclonal antibodies. Meanwhile, cellular protein C1QBP and viral protein IE2-p86 were detected with aforementioned monoclonal antibodies.

### Immunofluorescence assay

Cells on coverslips were washed twice in PBS, followed by fixation in 4% paraformaldehyde for 20 min at room temperature. After rinsing with PBS, cells were permeablized with 0.3% Triton X-100 at room temperature for 5 min. For analysis of nuclei, cells on coverslips were permeablized with 0.3% Triton X-100 for 5 min before fixation with 4% paraformaldehyde for 10 min at room temperature. The cells were washed three times with PBS prior to being blocked with 2% bovine serum albumin and 0.01% Tween-20 for 30 min. The cells were incubated with primary antibodies described above in blocking buffer at 4°C overnight. After extensive washing with PBS, the coverslips were incubated for 60 min with secondary antibody IgG2A-specific Alexa Fluor 568-coupled antibodies (Ab) or IgG-specific Alexa Fluor 488-coupled Ab (Invitrogen). Before mounting on slides, cells were incubated with either DAPI or ToPro-3 to stain cellular DNA. Samples were examined and photographed under an Olympus DX-51 fluorescence microscope or a Zeiss 510 confocal microscope. In addition, Mitotracker Red dye (Molecular Probe) was used to show cell mitochondria. To verify the colocalization of viral proteins and cellular proteins, confocal microscopy photos were analyzed for Pearson colocalization coefficient (R) by ImageJ (ver. 1.46).

### Immunoprecipitation

HFF cells were infected with wt Towne virus at MOI of 2 PFU/cell for 48 h before immunoprecipitation assay. 2×10^6^ infected cells were lysed by freeze-thaw cycle in NP-40 lysis buffer containing protease and phosphatase inhibitors (50 mM Tris-HCl [pH 8.0], 125 mM NaCl, 1 mM EDTA, 50 mM NaF, 1 mM Na_3_VO_4_, 1 mM DTT, 1 mM PMSF, 0.5% NP-40, and 1× protease inhibitor cocktail). Cell lysates were pre-cleared with protein G agarose beads (Pierce, Rockford, IL) and incubated with 2 µg of antibody plus 50 µl of protein G agarose beads at 4°C overnight. Antibodies used in this study are described above. Purified mouse IgG1 (ab27479, Abcam) and normal rabbit serum were used as isotype control. Immunoprecipitates were washed four times with NP-40 washing buffer (50 mM Tris-HCl [pH 8.0], 125 mM NaCl, 1 mM EDTA, 50 mM NaF, 1 mM Na_3_VO_4_, 1 mM DTT, 1 mM PMSF, 0.1% NP-40), and then suspended in an equal volume of 2X SDS-PAGE loading buffer (200 mM Tris-HCl [pH 7.8] containing 8% SDS, 0.02% bromophenol blue, and 20% β-mercaptoethanol). Samples were fractionated by 10% SDS-PAGE and transferred to PVDF membranes. Western blot analysis was performed as described previously [27], [31].

### Expression of IE2-p86 or GS-IE2-p86 protein in HFF cells

Replication-defective E1a^−^, E1b^−^, and E3^−^ recombinant adenovirus vectors expressing either the tetracycline-inducible “Tet-off” transactivator (AdTrans), the wildtype IE2-p86 protein (Ad-IE86), or IE2-p86 protein with N-terminal GS tag (Ad-GSIE86) were generated by Gene Transfer Vector Core at the University of Iowa as described previously [13], [27]. The titers of the various recombinant adenovirus vectors were determined by plaque assay on 293 cells. For transduction experiments, 1×10^8^ HFF cells were transduced by Ad-IE86 or Ad-GSIE86 at MOI of 20 PFU/cell in the presence of AdTrans and Lipofectamine 2000 reagent (3 µl/ml medium, Invitrogen, Carlsbad, CA) in Eagle's MEM media. After 48 h, the cells were harvested for TAP procedure.

## Results

### Generation and identification of Towne IE2SG recombinant viruses

To identify proteins associated with HCMV IE2-p86 in virus-infected cells, we generated recombinant viruses expressing IE2-p86 protein fused with a SG tag for tandem affinity purification procedure using a two-step *RED* recombination method. Figure 1B diagrams the recombination procedure as described in the Materials and Methods. Spurious recombination events of wild type (wt) and recombinant BAC clones were not detected by DNA sequencing or RFLP analysis with restriction endonucleases EcoRI and Hind III (Fig. 1C). wt and mutant recombinant viruses were isolated from HFF cells transfected with BAC DNAs and were designated as Towne-wt, IE2SG1, and IE2SG2.

**Figure 2:**
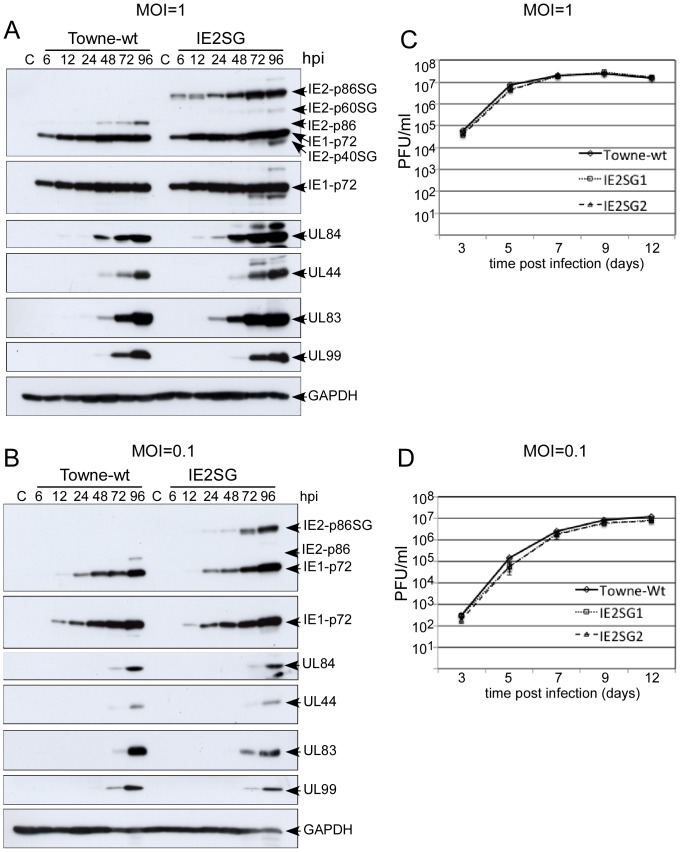
Growth property of HCMV recombinant virus IE2-p86SG. Expression of viral proteins in HFF cells infected with either wt Towne or IE2SG (A and B). HFF cells were infected at high (1 PFU/cell) or low (0.1 PFU/cell) MOI and analyzed by Western blot analysis. MIE viral proteins IE1-p72, IE2-p86, IE2-p86SG, early viral proteins UL84 and UL44, early/late viral proteins UL83 and late viral protein UL99, and cellular protein GAPDH were detected using specific antibodies as described in the Materials and Methods. (C and D) Growth curves of wt HCMV BAC Towne and Towne-IE2SG after high (1 PFU/cell) or low (0.1 PFU/cell) MOI. Cell culture supernatants were harvested at various d p.i. for viral titers via a GFP-based plaque assay. The standard deviations indicated for each data point on the graphs were derived from three independent experiments.

First, we compared the kinetics of viral protein accumulation and the growth properties of Towne-wt to the mutant recombinant viruses. HFF cells were infected in parallel with Towne-wt and the IE2SG mutant viruses at both high (1) and low (0.1) MOIs. At various times p.i., cells were harvested for immunoblotting with specific antibodies against viral IE (UL122 and UL123), E (UL44 and UL84), and L (UL83 and UL99) proteins. At an MOI of 1.0, IE1-p72 and IE2-p86 proteins were detected with the expected molecular weight in Towne-wt infected HFF cells. In cells infected with IE2SG, the IE2-p86 protein migrated as a larger protein (110 kDa), consistent with the SG tag, while the IE1-p72 was not affected (top panel in Fig. 2A). In addition, all examined viral proteins showed comparable expression dynamics in Towne-wt- and IE2SG-infected cells, with the exception of proteins expressed from the IE2 gene locus (Fig. 2A). The slightly higher quantities of UL84 and UL83 with IE2SG compared to Towne-wt may be due to a slight difference in actual MOI. The SG tag has two copies of Protein G peptide, which tightly binds to the Fc portion of antibodies. Therefore, IE2SG will be detected with high sensitivity in Western blot assay. For the same reason, the other two IE2 proteins expressed during late phase, IE2-p60 and IE2-p40, were also detected in IE2SG-infected cells (top panel in Fig. 2A). At low MOI, Towne-wt and the IE2SG recombinant viruses had almost identical expression patterns of IE1-p72, UL84, UL44, UL83, and UL99 proteins (Fig. 2B). Protein expression data demonstrated that the addition of SG tag to IE2-p86 did not affect HCMV protein expression.

The two independent recombinant viruses, IE2SG1 and IE2SG2, exhibited the same growth curves under conditions of both high and low MOI compared to that for the wt virus (Fig. 2C and D). Taken together, these results indicate that fusion of the SG tag to the C-terminus of IE2-p86 protein does not affect HCMV replication nor the function of IE2-p86 protein at both high and low MOI. Thus, all the major functions of the IE2-p86 protein are likely preserved in the mutant virus IE2SG. This allowed us to purify protein complexes associated with IE2-p86 protein during the course of HCMV infection in fibroblast cells.

### Identification of proteins associated with SG-tagged IE2-p86 in infected cells by LC-MS/MS

**Figure 3:**
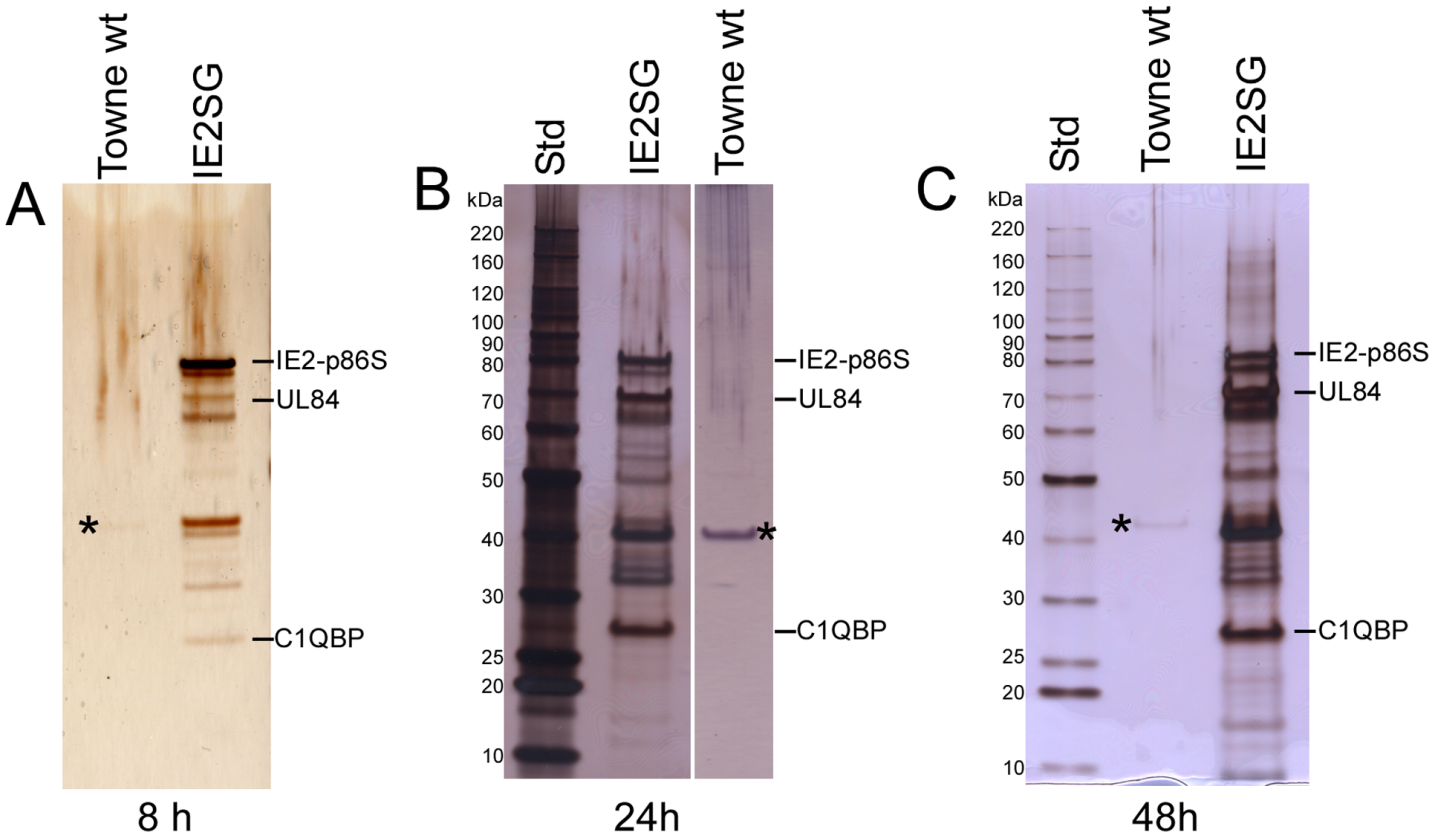
TAP of protein complexes associated with IE2-p86SG. 1×10^8^ HFF cells were infected in parallel with wt HCMV Towne or IE2SG at an MOI of 2 PFU/cell, and harvested at 8 (A), 24 (B), and 48 (C) h p.i. TAP with IgG sepharose resin and Strepavidin Sepharose resin was as described in the Materials and Methods. One tenth of the purified eluate was fractionated by SDS-PAGE, and visualized by silver staining. IE2-SBP indicates the tagged IE2-p86 protein with the IgG binding moiety removed by TEV protease. The position of viral protein UL84 and cellular protein C1QBP in the gel are also designated. The protein standard (Std) represents approximately 50 ng of protein per band. The asterisk marks the sole protein band (beta-actin) in the Towne wt sample that is visible on the silver staining gel.

To identify proteins associated with SG-tagged IE2-p86 in infected cells, a series of tandem affinity purifications were performed as described previously [28]. The purified protein complexes from cells infected with Towne-wt or IE2SG (1/10 of the final eluates) at 8, 24, and 48 h p.i., were fractioned by gradient SDS-PAGE and visualized by silver staining to monitor the purification quality. A variety of proteins co-purified with IE2-p86SG protein from IE2SG-infected cell lysates at all three time-points, but only one protein band (around 40 kDa) was visible with Towne-wt infected cell lysates at 8, 24, and 48 h p.i. and it was identified by MS as beta-actin (Fig. 3A, B, and C). At 8 h, the most abundant protein, which migrated between 80 and 90 kDa, was the bait protein IE2-p86 fused with the SBP moiety but lacking the ProtG moiety (IE2-p86S) as confirmed by Western blot analysis (Fig. S1). The IE2-p86SG associated proteins of all three time-points shared a similar pattern as to number and size of bands. However, the samples from later time-points had a greater quantity of protein bands. These data indicate that the proteins associated with IE2-p86SG were enriched through the TAP procedure.

The remaining biotin eluates of IE2SG and the controls at three time-points were digested by trypsin, and the resulting tryptic peptides were recovered by a reversed-phase chromatography (RPC) column and analyzed by LC-MS/MS as described in Materials and Methods. Data generated by LC–MS/MS were searched against SwissProt protein database with both Mascot and X! Tandem programs for identification of proteins in the IE2-p86 associated complexes. Only cellular and viral proteins that were co-purified from IE2SG infected lysates were considered. After disgarding findings of common contaminants of AP-MS experiments (i.e. keratin, trypsin, myosin and actins) [36] a total of 84 proteins were identified in IE2SG samples with high confidence (>99%), as based on the criteria that at least 2 high quality unique peptides (>95%) matched to the same protein (see Table 2 and 3). As expected, the bait protein IE2-p86 was abundant in all samples obtained at 8, 24, and 48 h p.i. UL84, the early viral protein that is known to bind to IE2-p86 [24], [38], was also abundant in all three samples. Since IE2-p86 and UL84 proteins are the only two viral proteins identified in the 8 h sample (Table 2) and it is known that they interact [25], [39], it is conceivable that UL84 directly associated with IE2-p86 very early and the association existed throughout the first 48 h after infection. At 24 h p.i., additional viral proteins UL44, UL24, and UL112 were detected in the IE2SG sample. Four more viral proteins (UL25, UL83, UL29, and IRS1) were present in the 48 h sample. These findings suggest that additional viral proteins come into association with IE2-p86 in accordance with the temporal order of expression during the viral replication cycle. Except for UL24 and UL29, all viral proteins shown in Table 2 were also identified in previous MS-based studies using UL84 or UL44 as bait [22], [24]. These data further confirm the success of TAP using IE2-p86 as bait protein and MS/MS analysis. The viral protein UL97 was previously reported to be associated with UL44 [40], [41], but we, and others by AP and immunoprecipitation [22], [42] did not detect UL97. UL97 may be an example of proteins that interact transiently and are not detected as stable and abundant protein complexes. We also did not detect UL54, which is a part of the HCMV core replication machinery [25]. Of the 75 cellular proteins, 13 proteins are present in IE2SG samples at all three time-points. These IE2-p86-associated proteins included polymerase I and transcript release factor (PTRF-1), complement component 1 Q subcomponent-binding protein (C1QBP), nuclear phosphoprotein B23 (NPM1), and nuclease-sensitive element-binding protein 1 (YBX1). Several cellular proteins were previously shown to associate with UL84 or UL44 [22], [42], such as components of importin (Importin alpha-3 and alpha-4), casein kinase (CSNK2B, CSNK2A1, and CSNK2A2), and nucleolin. At all three time-points, peptides of various ribosomal proteins were identified. HCMV infection significantly upregulates host rRNA and increases the total number of cellular ribosomes [43]. We did not identify any ribosomal proteins in TAP eluates of cells over-expressing IE2-p86 protein fused with N-terminal GS tag from a replication defective adenoviral vector (Fig. S2 and Table S1). The ribosomal proteins were associated with IE2-p86 in the presence of other viral or cellular proteins and have been detected in other systems [36], [44].

**Table 2:** Proteins identified from MS/MS analysis of IE2-p86SG associated complex.

		Accession Number	MW (kD)	No. of Unique Peptide			Sequence Coverage 48 h (%)
	Protein Name			8 h	24 h	48 h	
	Regulatory protein IE2 (UL122)	VIE2_HCMVT	63	23	41	27	47.0
	Protein UL84 (UL84)	UL84_HCMVT	65	20	35	29	57.0
	DNA polymerase accessory protein (UL44)	A9YU14_HCMV	46		3	14	55.0
Viral	Tegument protein UL24 (UL24)	B8YE60_HCMV	34		6	12	51.0
Protein	Protein UL112 (UL112)	D2K5K1_HCMV	70		5	8	23.0
	Tegument UL25 (UL25)	A8T788_HCMV	74			8	18.0
	Protein UL29 (UL29)	D3YRW8_HCMVT	79			5	10.1
	Tegument protein pp65 (UL83)	PP65_HCMVT	63			3	7.7
	Tegument protein IRS1 (IRS1)	E7DVS9_HCMV	93			2	3.1
	Importin alpha-3 (KPNA3)	Q8IYQ9_HUMAN	58		16	11	43.0
	Importin alpha-4 (KPNA4)	IMA4_HUMAN	58		8	9	49.0
	Casein kinase 2 beta polypeptide (CSNK2B)	B0UXA9_HUMAN	25		8	8	57.0
	Casein kinase II subunit alpha (CSNK2A1)	E7EU96_HUMAN	45		11	13	45.0
	Casein kinase II subunit alpha' (CSNK2A2)	CSK22_HUMAN	41		7	9	37.0
	Complement component 1 Q subcomponent-binding protein (C1QBP)	C1QBP_HUMAN	31	11	10	18	63.0
	nucleolar phosphoprotein B23 (NPM1)	NPM_HUMAN	30	4	7	5	36.0
	Polymerase I and transcript release factor (PTRF-1)	PTRF_HUMAN	42	3	9	6	19.0
	Nuclease-sensitive element-binding protein 1 (YBX1)	YBOX1_HUMAN	30	5	5	10	61.0
	Neocleolin (NCL)	E7EX81_HUMAN	66			10	21.0
	Histone-binding protein (RBBP4)	RBBP4_HUMAN	48			5	22.5
Cellular	Retinoblastoma binding protein 7 (RBBP7)	RBBP7_HUMAN	46		2		
Protein	Polyadenylate-binding protein 1 (PABPC1)	PABP1_HUMAN	71		2	4	8.1
	Heterogeneous nuclear ribonucleoprotein U (HNRNPU)	B4DLR3_HUMAN	87		4	5	9.6
	Heterogeneous nuclear ribonucleoprotein M (HNRNPM)	HNRPM_HUMAN	78			2	6.3
	Nucleolar RNA helicase 2 (DDX21)	DDX21_HUMAN	87			4	3.6
	ATP-dependent RNA helicase DDX17 (DDX17)	DDX17_HUMAN	73			3	6.0
	Death associated protein 3 (CRA3)	B4DP59_HUMAN	41		2		
	Eukaryotic translation initiation factor 6 (EIF6)	IF6_HUMAN	35			2	30.0
	Purine-rich element binding protein A (PURA)	Q2NLD4_HUMAN	32			2	21.0
	Heparin-binding protein HBp15 (HBp15)	Q7Z4W8_HUMAN	15			2	38.6
	Cell growth-inhibiting protein 34	Q08ES8_HUMAN	20			2	9.6
	Tripartite motif-containing 26 (TRIM26)	Q5SRL2_HUMAN	62			3	8.0
	2,4-dienyl-CoA reductase	DECR_HUMAN	35		3	3	14.0
	CSDA protein	Q96GD7_HUMAN	32			4	29.0
	78 kDa glucose-regulated protein (HSPA5)	GRP78_HUMAN	72		9	15	32.0
	Histone H2A (HIST1H2AH)	H2A1H_HUMAN	14			3	29.0
	Histone H1.2 (HIST1H1C)	H12_HUMAN	21		4	5	16.0
	Histone H1.5 (HIST1H1B)	H15_HUMAN	23		3	2	12.0

Table 2 and 3 are showing representative data from 2, 1, and 3 repeats of 8, 24, and 48 h samples, respectively. Proteins that uniquely identified in HCMV IE2SG infected cells after removal of common contaminants. Ribosomal proteins are excluded from this list, and are shown in Table 3. Each protein (probabilty >99%) shown here has at least two unique peptides indentified by MS/MS anlysis, and each peptide with >95% confidence. Note:

**Table 3:** Ribosomal proteins indentified in IE2-p86SG associated complex.

				No. of Unique Peptides		
	Protein Name	Uniprot Accession Number	MW(kD)	8 h	24 h	48 h
	**RPLP0**	A8K4Z4_HUMAN	34		3	6
	RPLP1	RLA1_HUMAN	11			2
	**RPLP2**	RKA2_HUMAN	12	3	4	6
	**RPL3**	RL3_HUMAN	46	3	7	11
	**RPL4**	RL4_HUMAN	46		6	12
	**RPL6**	RL6_HUMAN	33		6	6
	RPL7	RL7_HUMAN	24		3	3
	RPL7A	RL7A_HUMAN	30	3	5	6
	**RPL8**	RL8_HUMAN	21	3	5	4
	RPL10	RL10_HUMAN	27		2	4
	RPL10a	RL10A_HUMAN	25			3
	RPL11	RL11_HUMAN	20			2
	RPL12	RL12_HUMAN	18		2	2
	RPL13	RL13_HUMAN	24	3	3	3
60S	RPL13A	RL13A_HUMAN	24		3	2
subunit	**RPL14**	RL14_HUMAN	24		4	5
	RPL17	RL17_HUMAN	21		5	4
	RPL18	RL18_HUMAN	22			4
	RPL18a	RL18A_HUMAN	21		2	2
	RPL19	RL19_HUMAN	23		2	2
	RPL21	RL21_HUMAN	19			2
	RPL23	RL23_HUMAN	12			2
	RPL23A	RL23A_HUMAN	22		4	3
	RPL24	RL24_HUMAN	18		2	4
	RPL26	RL26_HUMAN	18		3	3
	RPL27	RL27_HUMAN	16	2	2	2
	RPL27A	RL27A_HUMAN	10			3
	RPL30	RL30_HUMAN	13			2
	RPL31	RL31_HUMAN	14	2	2	2
	RPL32	RL32_HUMAN	18		3	4
	**RPS2**	RS2_HUMAN	31		4	5
	**RPS3**	RS3_HUMAN	27		4	5
	RPS3A	RS3A_HUMAN	30		4	6
	RPS4X	B2R491_HUMAN	30		2	2
	**RPS6**	RS6_HUMAN	29	2	3	4
	**RPS7**	RS7_HUMAN	22			2
	**RPS8**	RS8_HUMAN	24	5	7	5
40S	RPS9	RS9_HUMAN	23		2	3
subunit	RPS15	RS15_HUMAN	17			2
	RPS15a	RS15A_HUMAN	15			4
	RPS12	RS12_HUMAN	15		4	3
	RPS14	RS14_HUMAN	16		3	3
	RPS24	RS24_HUMAN	32			3
	RPS25	RS25_HUMAN	14		3	3
	RPS26	RS26_HUMAN	13			2
	RPS27A	RS27A_HUMAN	18			2

Note: Representative ribosomal proteins used to draw protein interaction network are in bold and underlined.

### Protein-protein interaction network of IE2-p86 associated proteins in the context of HCMV infection

**Figure 4:**
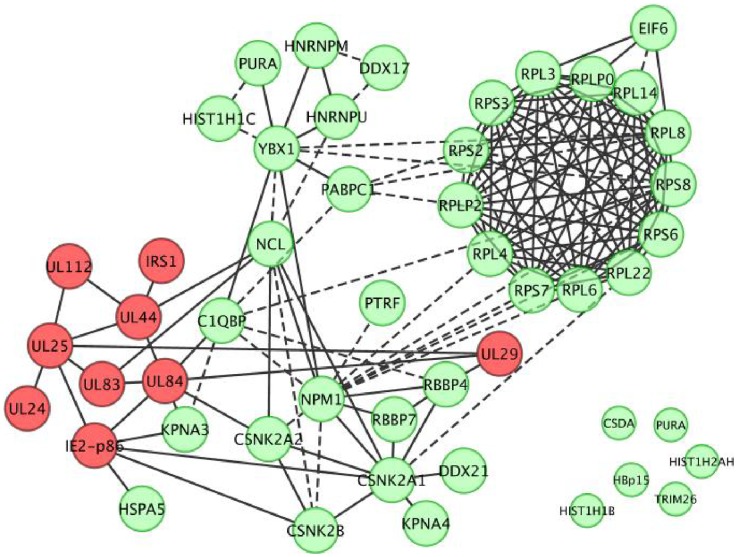
An interaction network of IE2-p86 associated proteins identified in HCMV IE2SG- infected HFF cells. To map the landscape of the IE2-p86 protein interactions, *in silico* networking analysis of identified proteins in both Table 2 and 3 using STRING database combined with published experimental data were schematically presented by Cytoscape software (version 2.8.3). Solid lines show physical protein–protein interactions based on experimental evidence from published reports and STRING search results. Dashed lines indicate the predicted protein–protein association by STRING database using predictive methods, such as textmining, coexpression, neighborhood, and co-occurance [37]. To simplify the map, only representative ribosomal proteins were shown.

To search for known evidence of physical/functional interactions between the various components of the IE2-p86 interactome identified by TAP-MS, a computational network analysis based on both online database (Search Tool for the Retrieval of Interacting Genes, STRING, Ver.9.0) and the bibliography was performed. To this end, all unique non-ribosomal proteins (Table 2) and 13 representative ribosomal proteins (Table 3) were searched using the STRING database. In addition, the protein-protein interaction data of those identified viral proteins was compiled from published research papers and was combined with the STRING database search result, which was then imported into Cytoscape software (version 2.8.3) for visualization. The resulting network of HCMV IE2-p86 interactome shown in Figure 4 is an overall landscape of potential interactions, without indication of the actual protein complexes. The first notable aspect of the network is the high-level interconnection of all nine viral proteins. While IRS1 and UL24 are reported to interact with only UL44 and UL25, respectively [45], [46], the other seven viral proteins have reported interactions with at least two other viral proteins, UL84, UL44 and UL25 each has at least four direct interacting partners. The majority of identified non-ribosomal cellular proteins are interconnected with NCL, NPM1, YBX1 and C1QBP as interaction hubs. The ribosomal protein complexes are connected to other cellular proteins through YBX1 and NPM1. The highly interconnected viral protein network connects to the cellular network through IE2-p86/UL84/UL44 and NCL/C1QBP/NPM1to form a large protein-protein interaction network. Taken together, the protein-protein interaction network suggests that IE2-p86 is a part of multiple viral and cellular protein interactions. However, the TAP-MS method demonstrated stable protein-protein interactions of abundant cellular proteins and not transitory interactions of less abundant proteins. Both type of interactions are important to the virus replication cycle.

### Immunoprecipitation of IE2-p86 viral and cellular protein complexes

**Figure 5:**
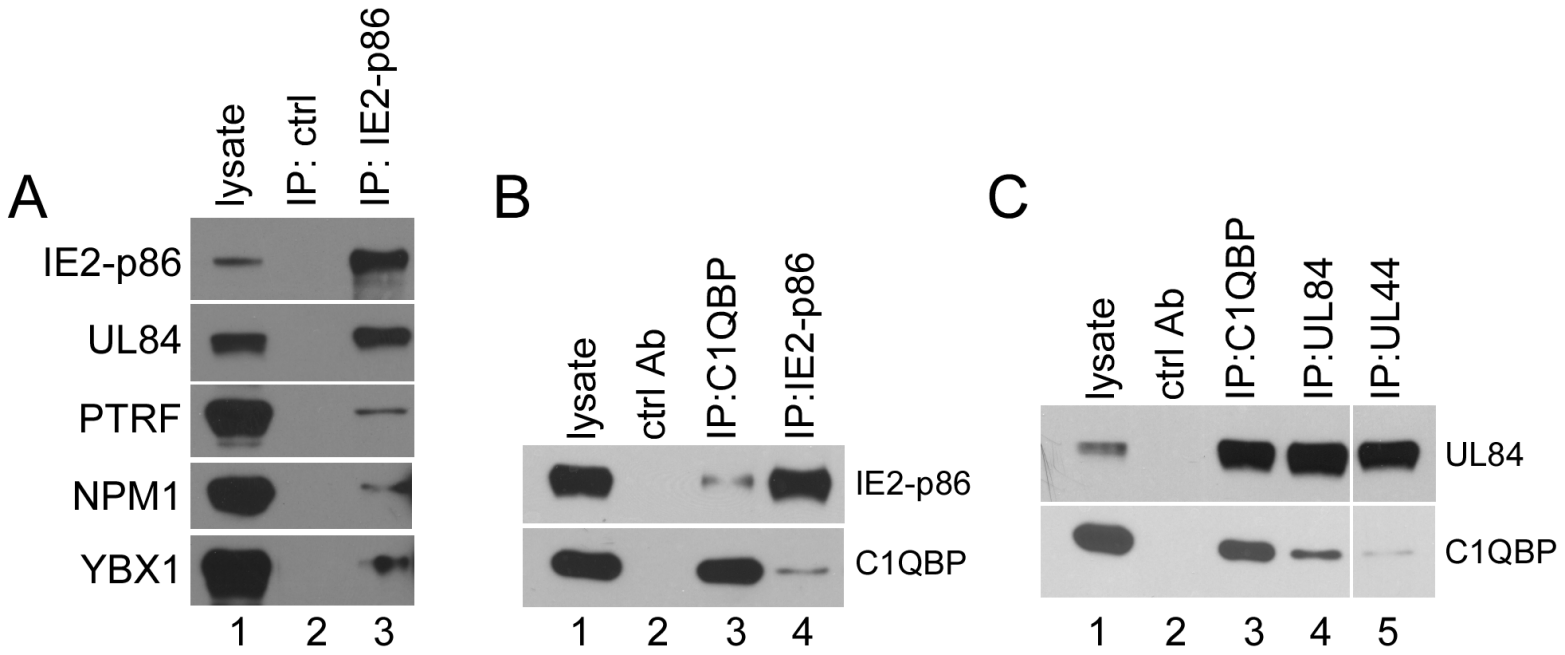
Association of cellular proteins with viral proteins IE2-p86, UL84 and UL44 in co-immunoprecipitation. HFF cells were infected with wt Towne at an MOI of 2/cell, harvested at 48 h p.i. for immunoprecipitation with specific antibodies against C1QBP (A, B and C), IE2-p86 (A and B), UL84 (C), UL44 (C), or isotype antibody control (ctrlAb). The precipitates were fractionated by 10% SDS-PAGE. Proteins were detected by Western blot with indicated specific antibodies (PTRF, NPM1, YXB1, and C1QBP) as described in the Materials and Methods. Since the heavy chain of the antibody used in co-immunoprecipitation migrates to the same location as UL44, and overshadows the signal of UL44, the protein UL84 was examined in anti-UL44 precipitates (C).

To verify the association of viral and cellular proteins with IE2-p86, Towne-wt-infected HFF cell lysates were immunoprecipitated with anti-IE2-p86 antibody and analyzed by Western blotting analysis. In agreement with TAP data and the literature [25], [39], viral proteins IE2-p86 and UL84 were detected in the immunoprecipitates with anti-IE2-p86 antibody, but not with isotype antibody control (Fig. 5A). Cellular proteins PTRF, NPM1, and YBX1 were also detected in the anti-IE2-p86 antibody immunoprecipitates but not with the isotype antibody controls (Fig. 5A, lanes 2 and 3). The cellular proteins associated with IE2-p86 were in relatively lower abundance than UL84. These data confirm the TAP-MS results that specific viral and cellular proteins interacted with IE2-p86 in HCMV-infected cells.

### Reciprocal Immunoprecipitation of C1QBP with IE2-p86 and UL84

To investigate further at least one of the viral-cellular protein complexes, we choose C1QBP (a.k.a p32, HABP1). First, we performed reciprocal immunoprecipitation using anti-C1QBP, -IE2-p86, -UL84 and -UL44 antibodies at 48 h p.i. As expected, C1QBP was precipitated with the anti-IE86 antibody (Fig. 5B, lane 4); and, conversely, the IE2-p86 protein was precipitated with the anti-C1QBP antibody (Fig. 5B, lane 3). C1QBP was also precipitated with anti-UL84 antibody (Fig. 5C, lane 4). The relative amount of UL84 immunoprecipitated by anti-C1QBP was at the same abundance as immunoprecipitated by anti-UL84 antibody (Fig. 5C, lane 3). C1QBP was also immunoprecipitated with the anti-UL44 antibody, but at a lower relative abundance (Fig. 5B, lane 5). Because UL44 and immunoglobulin G heavy chain both migrate to the same position in SDS-PAGE and the UL44 signal was overshadowed by that of antibody heavy chain, UL84 was probed in the anti-C1QBP immunoprecipitate instead. These data confirm the earlier observation that UL84 directly interacts with C1QBP [24]. Since UL84 directly interacted with IE2-p86 and C1QBP, respectively, while over-expressed IE2-p86 couldn't pull down C1QBP in the absense of UL84 (Fig. S2 and Table S1), it is likely that C1QBP associates with IE2-p86 indirectly through its interaction with UL84 [24].

### Nuclear co-localization of C1QBP and IE2-p86/UL84/UL44 complex

**Figure 6:**
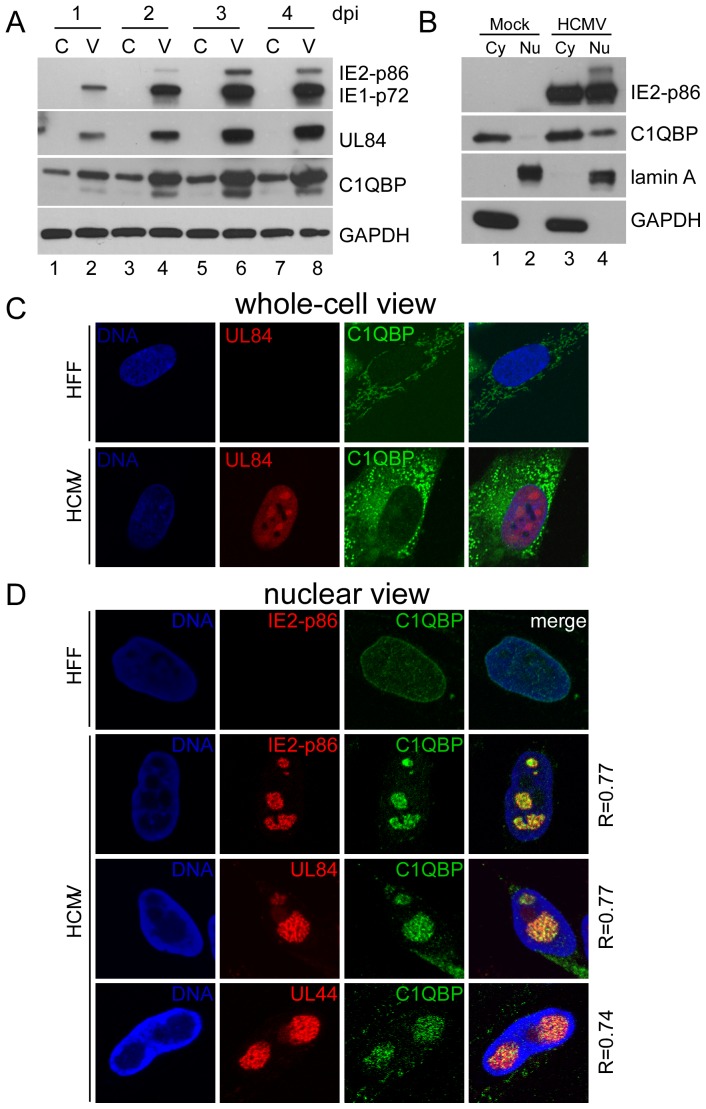
HCMV infection up-regulates the expression of cellular protein C1QBP, which associates with the IE2-p86/UL84/UL44 complex in virus replication compartments. (A) HFF cells, infected with wt HCMV (V) at an MOI of 2 PFU/cell and were harvested at the indicated time-points for western blot analysis of proteins IE1-p72, IE2-p86, UL84, C1QBP, and GAPDH by using specific antibodies described in the Materials and Methods. Cellular protein GAPDH serves as sample loading control (C). (B) Cytoplasmic and nuclear fractions of mock-infected or HCMV-infected cells were analyzed for C1QBP and IE2-p86. Lamin A and GAPDH were used as makers for the nuclear (Nu) and cytoplasm (Cy) fractions, respectively. (C and D) Subcellular localization of cellular C1QBP and viral UL84, IE2-p86, and UL44 proteins in HFF cells infected with wt HCMV at MOI of 2 PFU/cell for 48 h. Uninfected cells and infected cells were fixed before (C, whole-cell view) or after (D, nuclear view) permeabilization with 0.3% Triton X-100 and immunostained with specific antibodies against C1QBP, IE2-p86, UL84, or UL44 as described in the Materials and Methods. Cellular DNA was stained by TO-PRO. The 63× objective lense was used. Pearson's correlation (R) for colocalizaion of fluorescent signals was determined for indicated images by Image J software (ver 1.46) and are shown on the right of each panel.

Since HCMV infection upregulates cellular gene expression [13] and C1QBP is associated with the IE2-p86, UL84 and UL44 in HCMV-infected cells, we hypothesized that HCMV infection may affect C1QBP expression or its localization within the cell. Figure 6A shows that the relative levels of C1QBP increased one day after infection and accumulated to very high levels along the progression of viral infection through day four. In comparison, there was little change in C1QBP expression in mock-infected cells. Next, we determined the effect of HCMV infection on the cellular distribution of C1QBP. At 48 h p.i., mock- and HCMV-infected HFF cells were separated into cytoplasmic and nuclear fractions based on the distribution profile of protein markers lamin A (nuclear) and GAPDH (cytoplasm). In mock-infected cells, the majority of C1QBP was present in cytoplasmic fraction, with very little presence in the nuclear fraction. In HCMV-infected cells, C1QBP was more abundant than that of mock-infected cells, especially in the nuclear fraction (Fig. 6B), suggesting that HCMV infection not only dramatically increases C1QBP in the cytoplasm, but also in the nucleus of infected cells, where IE2-p86 and UL84 are located.

To determine if the nuclear C1QBP colocalizes with viral proteins (IE2-p86, UL84 and UL44) in the same nuclear compartments in infected cells, we used immunofluorescence assay and confocal microscopy. HFF cells, infected by Towne-wt (MOI = 2), were fixed and permeablized by Triton X-100. Consistent with previous reports [47]–[49], C1QBP was primarily localized in the cytoplasm of mock-infected HFF cells with a weak signal in the nucleus. In HCMV-infected HFF cells, the C1QBP protein was still predominantly located in the cytoplasm (Fig. 6C), and its expression level was significantly increased, which confirmed the Western blot analyses (see Fig. 6A and 6B). To better visualize the nuclear C1QBP without affecting the nuclear structure, cells were briefly treated with 0.3% Triton X-100 before the fixation to remove most of the cytoplasmic content as described previously [50]. A low level of C1QBP was detected in the nuclei of mock-infected HFF cells. In contrast, there was increased C1QBP signal in the nuclei of infected cells (Fig. 6D). Viral proteins IE2-p86, UL84, and UL44 were located in the virus replication compartments of the nucleus, consistent with previous reports [51]. Cellular protein C1QBP was also located in the virus replication compartments (Fig. 6D) as indicated by its co-localization with IE2-p86, UL84, and UL44. To validate the spacial relationship between C1QBP and the viral proteins, Pearson co-localization coefficient (R) by ImageJ (ver. 1.46) were determined as described previously [23] and all values (see figure 6D) indicated a strong association. Taken together, these data indicated that C1QBP was upregulated by HCMV infection and C1QBP colocalized with the viral proteins IE2-p86, UL84, and UL44 in the virus replication compartments. The other viral-cellular protein complexes described above will require further investigation.

## Discussion

HCMV IE2-p86 protein is an essential viral protein of which many important functions have been described, including interaction with a large array of viral and celluar proteins (reviewed in Stinski and Petrik, 2008 [6]). However, most of the binding partners previously detected used *in vitro* assays or over-expression of a specific protein of interest in various cell types. In this study, we sought to identify proteins that associate with IE2-p86 during HCMV infection and to develop an interactome network. To take full advantage of the power of TAP, we generated a recombinant virus that expresses the IE2-p86 protein fused with an SG tag at the carboxyl terminus called IE2SG. Western blot and growth curve analyses indicated that the SG-tag had no detectable effect on viral replication. This observation is in agreement with a previous report that a C-terminal eGFP tag did not disrupt the function of IE2-p86 protein [52]. Thus, we were able to identifiy proteins that associate with IE2-p86 protein under close-to-physiological conditions.

Using the TAP-MS method, we found that 9 viral proteins and 75 cellular proteins were specifically associated with the IE2-p86SG protein during the first 48 h p.i. Based on computational analysis and data gathered in our current study, an interaction network of viral and cellular proteins was generated (Fig. 4). The bait protein IE2-p86 associated with viral protein UL84. Both proteins were more abundant than any other viral and cellular proteins at all three time-points examined (8, 24, and 48 h p.i., see Table 2). Besides UL84, we also identified UL44, UL83, UL112, IRS1, UL24, UL25, and UL29 in association with the IE2-p86 protein in HCMV infected cells. The roles of UL84 and UL44 in viral replication have been described previously [25], while the roles of UL83, UL112, and IRS1 have been discussed and required further investigation [24], [46], [53]. UL112 could form a compex with UL84 and IE2-p86 through direct interaction with UL44; however, it doesn't form a complex with other replication core proteins [53]. The biological significance of the UL24 tegument protein's presence in the complex is intriguing considering its early presence and abundance, even though the protein has been classfied as non-essential for HCMV replication [54], [55]. Using co-immunoprecipitation and yeast two hybrid assay, To et al. [65] confirmed that UL25 directly interacts with both IE2-p86 and UL24. In addition, UL29 associates with UL84 and UL25, and promotes accumulation of IE RNA [19], [21], [56]

IE2-p86 and UL84 were a dominant association at early times after infection and throughout the virus infectious cycle. The fact that more viral proteins and a higher number of unique peptides were identified at later time-points reflects the progression of virus infection. It is not a surprise that all nine viral proteins showed variable interconnections in the interaction network. It is also clear, based on previous AP-MS studies [24], [46] and this study (Fig. 4 and Table 2), that UL84 and UL44 associate with IE2-p86 in the absense of other viral DNA replication core proteins, such as UL54, as described previously [19], [22], [42].

Of the 75 cellular proteins identified to associate with IE2-p86 in this study, some have been reported previously to associate with either UL84 or UL44 [22], [24]. For example, the multifunctional protein C1QBP was found to interact with UL84 independent of other viral proteins [24]. In this study, we found that C1QBP not only associates with IE2-p86 at all three time-points and but also was upregulated by HCMV infection (Fig. 6). Though the mechanism of C1QBP upregulation remains to be investigated, we speculate the transcription of C1QBP is increased by HCMV infection, like many other genes that were upregulated during the HCMV infection [13], [57]. We further confirmed that upon HCMV infection, there is significant accumulation of C1QBP in the nucleus besides the mitochondrion [49]. Previous studies have shown that C1QBP could translocate to the nucleus under certain conditions, such as association with viral proteins and treatment with chemical reagents [48], [58]–[60]. Although the mechanism remains to be uncovered, we speculate that UL84 might play a role in the nuclear accumulation of C1QBP in HCMV-infected cells based on the following observations: 1) Reciprocal co-immunoprecipitation support the report that C1QBP directly interacts with UL84 [24]; 2) C1QBP colocalized with IE2-p86, UL84 and UL44 in the virus replication compartments in the nucleus; 3) UL84 shuttles between nucleus and cytoplasm [61]; 4) HSV ICP27, a viral protein that shares some functional characteristics with HCMV UL84, induces nuclear accumulation of C1QBP in both infected and transfected cells [59]. It is also possible that C1QBP traffics to the nucleus after being phosphorylated by viral or cellular kinases. C1QBP phosphorylated by MAP kinase has been shown to translocate to the nucleus [48]. In this scenario, casein kinase II (CK II) that associates with, and phosphorylates UL84 may also phosphorylate C1QBP and affect its subcellular localization. Alternatively, UL84 may bind and retain C1QBP in the nucleus.

C1QBP has long been implicated in gene transcription and/or splicing. It has a stong transcription activation domain [62], [63], binds to basal transcription factor TFIIB [64] and interacts with transcription factor FOXC-1 [65]. In addition, two viral regulatory proteins, EBNA-1 and HIV Tat, also interact with C1QBP [64], [66]. We speculate that nuclear C1QBP might be involved in the complex transcription machinery that drives MIE gene expression from the viral genome. In addition, C1QBP may also play a role in viral DNA replication. There was stong *in vivo* evidence for the localization of C1QBP to *oriP* of EBV through its interaction with EBNA-1 [66]. Considering the association and co-localization of C1QBP with IE2-p86, UL84, and UL44 in the virus replication compartment, it will be interesting to determine how C1QBP affects HCMV *oriLyt*.

Besides C1QBP, casein kinase II (CK2) subunits (CSNK2A1, CSNK2A2, and CSNK2B) were also shown to interact with UL84 directly, and were required for *oriLyt*-dependent viral DNA replication [24], [42]. CK2 also phosphorylates nucleolin (NCL) and retinoblastoma binding protein 4 (RBBP4), RBBP7, and DDX21 *in vitro* [67]. Moreover, CSNK2A1 was shown to be associated with IE2-p86 in a TAP study [68]. Nucleolin associates with HCMV UL44 and is required to maintain the architecture of virus replication compartments [22], [69]. YBX1, a protein with variable functions such as transcription, splicing regulation, and translation regulation, has been found in different complexes [70], [71], and is associated with multiple proteins identified in this study, such as nucleolin, PABPC1, HNRNPU, NPM1, DDX21 [72]. Importin alpha-3 and -4 proteins were also shown to interact wth UL84, though they were not found to stably associate with UL44 [22], [24]. Another interesting observation of our study is the abundant ribosomal proteins associated with the IE2-p86. Even though ribosomal proteins are commonly found in AP-MS studies [36], [44], we did not identify any ribosomal proteins in the Towne-wt control, as well as Ad-GSIE2-p86 transduced HFF cells (Fig. S2 and Table S1). The known and predicted interactions between ribosomal proteins and other cellular proteins, such as NPM1, YBX1, PABPC1, and CSNK2A1, suggest that the ribosomal proteins may associate with IE2-p86 through these interactions. The biological function of ribosomal proteins in association with IE2-p86 is unclear, however, considering interplay between some ribosomal proteins and viral proteins [73], [74], it is possible that ribosomal proteins play a role besides protein translation during HCMV infection.

It is noted that most of the cellular proteins previously reported to interact with IE2-p86 (reviewed in [6]) were not confirmed by this study. A possible explanation is that the TAP procedure selects for abundant proteins and is too stringent for identifying proteins of low abundance that interact transiently or loosely with IE2-p86. A less stringent purification might be more suitable to identify those weak binding partners.

In conclusion, the interactome data presented here not only demonstrated the abundant and temporal development of protein complexes that associated with IE2-p86, but also provides a framework that will benefit future studies concerning the IE2-p86 protein complexes during HCMV infection. A more detailed understanding of how the IE2-p86 protein and other viral protein interaction networks are organized in the virus-infected cell may lead to novel antiviral therapies.

## Supporting Information

Figure S1 Western blotting of TAP samples. Samples collected from tandem affinity purification procedure of HFF cells infected with HCMV IE2-p86SG for 48 h were analyzed by western blot using IE2-p86-specific antibody (12E2, sc-69835, Santa Cruz Biotechnology, Santa Cruz, CA). Ratios of each fraction loaded on the SDS-PAGE gel for western blot were indicated at the bottom of figure.
(TIF)

Figure S2 TAP of protein complexes associated with GS-IE2-p86 protein. 1×10^8^ HFF cells were transduced in parallel with Ad-IE86 or Ad-GSIE2-p86 at an MOI of 20 PFU/cell, and harvested at 48 h p.i. TAP with IgG sepharose resin and Strepavidin Sepharose resin was as described in the Materials and Methods. One tenth (1/10) of the purified eluate was fractionated by SDS-PAGE, and visualized by silver staining. S-IE2-p86 indicates the N-terminal tagged IE2-p86 protein with the IgG binding moiety removed by TEV protease. The protein standard (Std) represents approximately 50 ng of protein per band. The asterisk marks the sole visible protein band in Ad-IE86 sample on silver staining gel.
(TIF)

Table S1 Proteins identified in TAP samples of Ad-IE86- or Ad-GSIE2-p86-transduced HFF cells by MS/MS analysis. Note: The data is from a representative sample of three repeats. Each protein (probabilty >99%) shown here has at least two unique peptides indentified by MS/MS anlysis, and each peptide with >95% confidence. No ribosomal proteins that were identified in HCMV-infected HFF cells were found in the samples.
(DOCX)
