# Supplementary figures and images for: Interaction Network of Proteins Associated with Human Cytomegalovirus IE2-p86 Protein during Infection: A Proteomic Analysis
Source: PLoS One. 2013 Dec 16;8(12):e81583. doi: 10.1371/journal.pone.0081583 (PMC3864812; doi:10.1371/journal.pone.0081583)

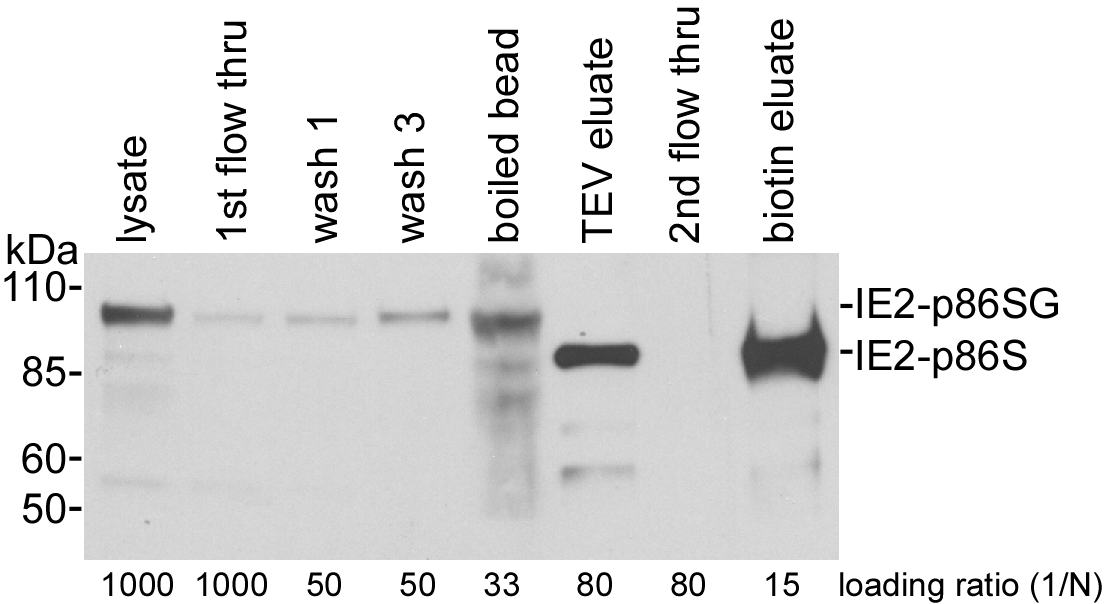

Supplement: Figure S1 — Western blotting of TAP samples. Samples collected from tandem affinity purification procedure of HFF cells infected with HCMV IE2-p86SG for 48 h were analyzed by western blot using IE2-p86-specific antibody (12E2, sc-69835, Santa Cruz Biotechnology, Santa Cruz, CA). Ratios of each fraction loaded on the SDS-PAGE gel for western blot were indicated at the bottom of figure. (TIF) [file pone.0081583.s001.tif]

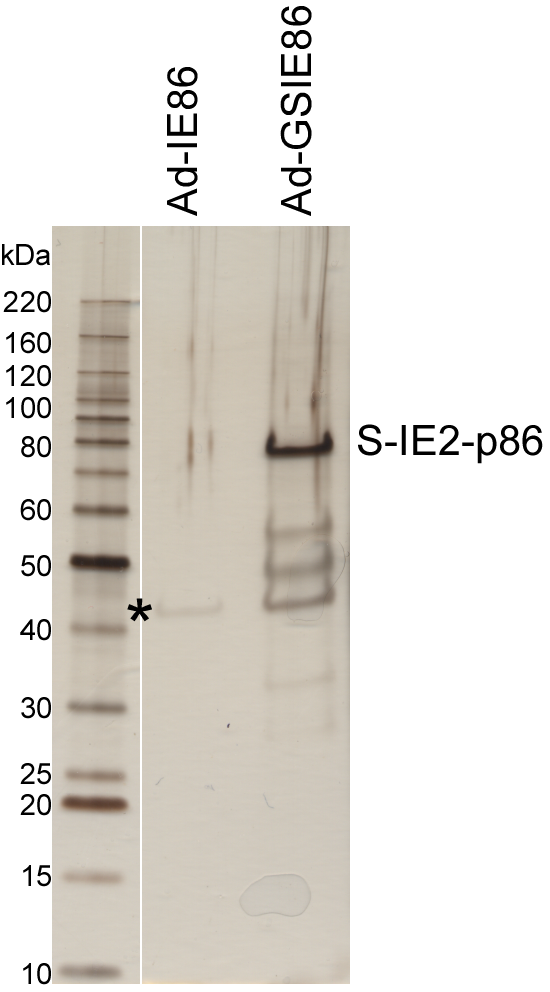

Supplement: Figure S2 — TAP of protein complexes associated with GS-IE2-p86 protein. 1×10^8^ HFF cells were transduced in parallel with Ad-IE86 or Ad-GSIE2-p86 at an MOI of 20 PFU/cell, and harvested at 48 h p.i. TAP with IgG sepharose resin and Strepavidin Sepharose resin was as described in the Materials and Methods. One tenth (1/10) of the purified eluate was fractionated by SDS-PAGE, and visualized by silver staining. S-IE2-p86 indicates the N-terminal tagged IE2-p86 protein with the IgG binding moiety removed by TEV protease. The protein standard (Std) represents approximately 50 ng of protein per band. The asterisk marks the sole visible protein band in Ad-IE86 sample on silver staining gel. (TIF) [file pone.0081583.s002.tif]
