# Supplementary material for: Interaction Network of Proteins Associated with Human Cytomegalovirus IE2-p86 Protein during Infection: A Proteomic Analysis
Source: PLoS One. 2013 Dec 16;8(12):e81583. doi: 10.1371/journal.pone.0081583 (PMC3864812; doi:10.1371/journal.pone.0081583)
Supplement: Table S1 — Proteins identified in TAP samples of Ad-IE86- or Ad-GSIE2-p86-transduced HFF cells by MS/MS analysis. Note: The data is from a representative sample of three repeats. Each protein (probabilty >99%) shown here has at least two unique peptides indentified by MS/MS anlysis, and each peptide with >95% confidence. No ribosomal proteins that were identified in HCMV-infected HFF cells were found in the samples. (DOCX) [file pone.0081583.s003.docx]

| Protein Name | Uniprot Accession Number | MW  (kD) | | No. Of Unique Peptides | | | |
| --- | --- | --- | --- | --- | --- | --- | --- |
|  |  |  |  | IE2-p86 | | GS-IE2-p86 | |
| Regulatory protein IE2 (UL122) | VIE2_HCMVT | | 63 | | 0 | | 18 |
| Importin alpha-3 (KPNA3) | Q8IYQ9_HUMAN | | 58 | | 0 | | 7 |
| Trypsin (pig) | TRYP_PIG | | 24 | | 5 | | 6 |
| 78 kDa glucose-regulated protein (HSPA5) | GRP78_HUMAN | | 72 | | 0 | | 5 |
| Beta actin | ACTB_HUMAN | | 42 | | 3 | | 5 |
| Keratin, type II cytoskeletal | K2C1_HUMAN | | 65 | | 3 | | 2 |
| Calmodulin | CALM_HUMAN | | 17 | | 0 | | 2 |
| Keratin, type I cytoskeletal 10 | K1C10_HUMAN | | 59 | | 2 | | 4 |
